# Validation of an accelerometer system for measuring physical activity and sedentary behavior in healthy children and adolescents

**DOI:** 10.1007/s00431-023-05014-z

**Published:** 2023-06-01

**Authors:** Camilla Milther, Lærke Winther, Michelle Stahlhut, Derek John Curtis, Mette Aadahl, Morten Tange Kristensen, Jette Led Sørensen, Christian Have Dall

**Affiliations:** 1 Juliane Marie Centre and Mary Elizabeths Hospital, Rigshospitalet, Copenhagen, Denmark; 2 Center for Clinical Research and Prevention, Copenhagen University Hospital, Bispebjerg and Frederiksberg, Denmark; 3 Child Centre Copenhagen, The Child and Youth Administration, City of Copenhagen, Denmark; 4 Institute of Clinical Medicine, University of Copenhagen, Copenhagen, Denmark; 5 Department of Physical and Occupational Therapy, Copenhagen University Hospital, Bispebjerg and Frederiksberg, Denmark

**Keywords:** Validity, Accelerometer, Children, Adolescents, Physical activity, Sedentary behavior

## Abstract

The study aims to assess the concurrent validity of the SENS motion^®^ accelerometer system for device-based measurement of physical activity and sedentary behavior in healthy children and adolescents. Thirty-six healthy children and adolescents (mean ± standard deviation (SD) age, 10.2 ± 2.3 years) were fitted with three SENS sensors while performing standardized activities including walking, fast walking, sitting/lying, and arm movements. Data from the sensors were compared with video observations (reference criteria). The agreement between SENS motion^®^ and observation was analyzed using Student’s *t*-test and illustrated in Bland–Altman plots. The concurrent validity was further evaluated using intraclass correlation coefficient (ICC) and was expressed as standard error of measurement (SEM) and minimal detectable change (MDC). Strong agreement was found between SENS and observation for walking time, sedentary time, and lying time. In contrast, moderate agreement was observed for number of steps, sitting time, and time with and without arm movement. ICC_2.1_ values were overall moderate to excellent (0.5–0.94), with correspondingly low SEM% for walking time, sedentary time, lying time, and time with arm movement (2–9%). An acceptable SEM% level was reached for both steps and sitting time (11% and 12%). For fast walking time, the results showed a weak agreement between the measurement methods, and the ICC value was poor.

*Conclusion*: SENS motion^®^ seems valid for detecting physical activity and sedentary behavior in healthy children and adolescents with strong agreement and moderate to excellent ICC values. Furthermore, the explorative results on arm movements seem promising.

**Table:** 

**What is Known:** *• Inactivity and sedentary behavior follow an increasing trend among children and adolescents.* *• SENS motion*^*®*^ *seems to be valid for measuring physical activity and sedentary behavior in adults and elderly patients.*	
**What is New:** *• SENS motion*^*®*^ *seems valid with strong agreement between video observations and SENS measurement, and ICC values are moderate to excellent when measuring physical activity and sedentary behavior in healthy children and adolescents.* *• SENS motion*^*®*^ *seems promising for detection of arm movements.*

**What is Known:**

*• Inactivity and sedentary behavior follow an increasing trend among children and adolescents.*

*• SENS motion*^*®*^ *seems to be valid for measuring physical activity and sedentary behavior in adults and elderly patients.*

**What is New:**

*• SENS motion*^*®*^ *seems valid with strong agreement between video observations and SENS measurement, and ICC values are moderate to excellent when measuring physical activity and sedentary behavior in healthy children and adolescents.*

*• SENS motion*^*®*^ *seems promising for detection of arm movements.*

## Introduction

Physical inactivity is a growing problem worldwide, also in the pediatric population. Most children and adolescents do not meet the minimum recommendations of a mean 60 min of daily physical activity of moderate to vigorous intensity (MVPA) [1, 2]. A recent systematic review found that children and adolescents (2–18 years) across 18 European countries reached a daily level of only ≤ 17 min of MVPA [3]. Furthermore, it appears that physical activity level decreases with age, and a simultaneous increase is observed in sedentary time among children and adolescents with age [4]. Interventions aiming to reduce sedentary behavior and increase physical activity levels in children and adolescents are important and require knowledge of their activity behavior. Thus, validated measurement tools to monitor physical activity levels and evaluate such interventions are needed. Physical activity is a complex human behavior, especially in children whose whole body is often involved in their movement patterns. We are therefore interested in identifying activities that comprise both the lower and upper extremity, of which arm movement is still exploratory.

Accelerometry is a device-based method to assess sedentary behavior and specific types of physical activity, e.g., walking, standing, and number of steps [5]. It is frequently used in clinical and research settings for measuring intensity counts per minute and body positions, allowing for estimation of the duration, intensity, and frequency of specific activity types [6]. Validation of device-based measurements, such as ActiGraph, Fitbit, and activPAL, has previously been established [7]. A recently developed accelerometer system, The SENS motion^®^ system (SENS), has beneficial features compared with similar devices. SENS has a longer battery lifetime than other systems and improved data storage capabilities owed to continuous data transfer by a wireless smartphone network connection, thereby reducing the risk of data loss. Furthermore, SENS has a more user-friendly output since data are transferred directly through a smartphone application. In previous studies, SENS has been validated in adult patient populations with promising results, except for the recording of steps in patient with slow walking speed [8, 9].

Children’s movements differ from those of adults as children move more sporadically and with rapid changes in the intensity of their activities, e.g., during play [10, 11]. Thus, it seems highly relevant for clinicians and researchers alike to validate this device in the pediatric population for measuring physical activity and sedentary behavior. Since SENS has not previously been validated in a pediatric population, the purpose of this study was to evaluate the concurrent validity of the SENS motion^®^ system in healthy children and adolescents by determining its ability to measure time spent being physically active and in sedentary behavior compared with video observations in a cross-sectional study.

## Materials and methods

### Study design and participants

This cross-sectional study aimed to assess the concurrent validity of the SENS accelerometry system compared with video observation. The participants were recruited from two randomly selected schools in the Capital Region of Denmark. Using convenience sampling, we invited pupils from the 1st and 3rd grade from one school and pupils from the 6th grade from the second school. The inclusion criteria were age between 7 and 13 years and no injuries or disabilities preventing the children from walking. The parents of participating children received an information letter about the study including a consent form a minimum of 2 weeks prior to the test day. All pupils who met the inclusion criteria and had a parent-signed consent form were included in the analysis.

### Data collection

**Fig. 1:**
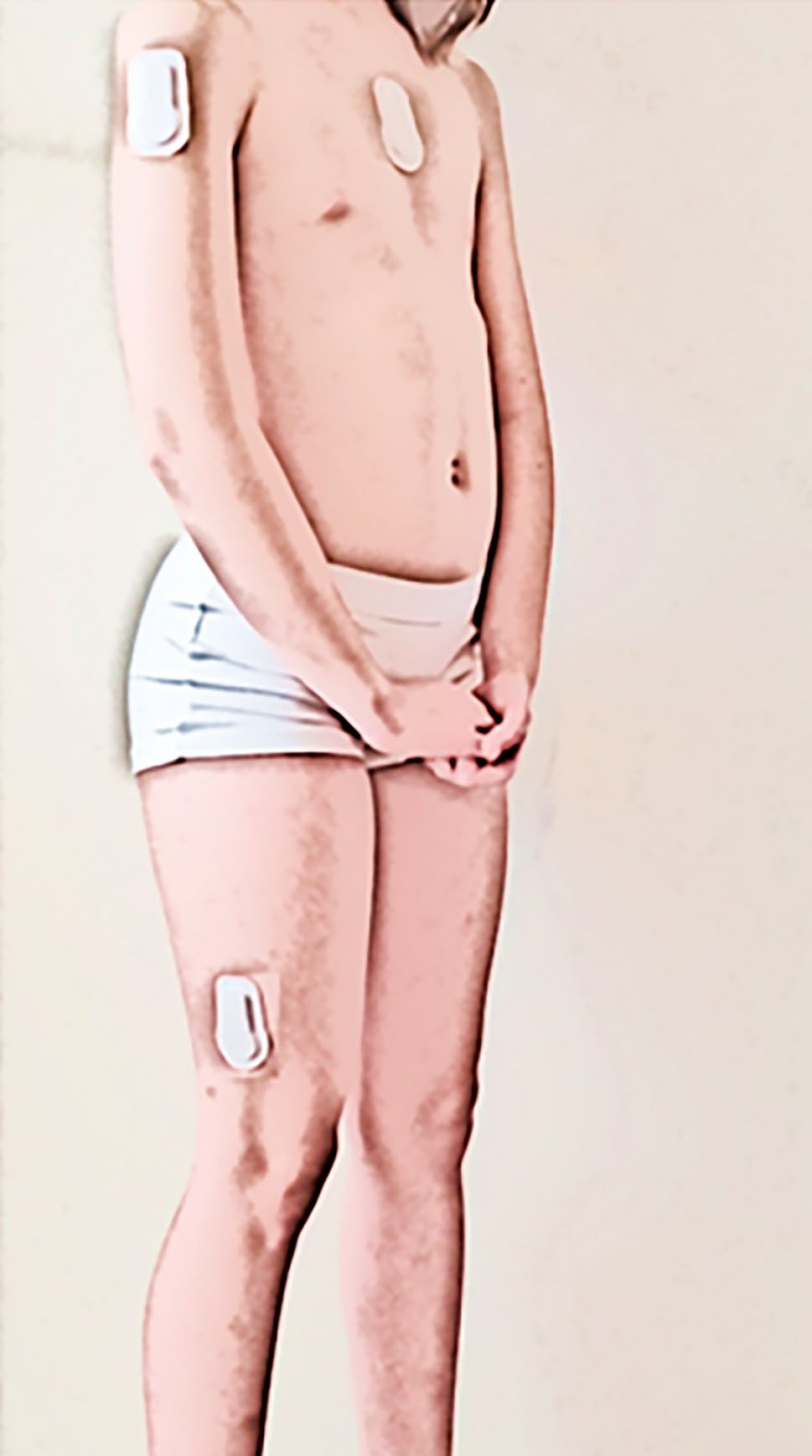
Placement of the three SENS sensors

Two of the authors were responsible for inclusion, data collection, and assessment (C. M., M. S.). Data collection was conducted in 3 days, and each class participated on one of the days. Information on age and sex was registered, whereas height was measured using tape measure and weight measures were using a Withings digital scale. Each participant was fitted with three SENS sensors placed on the thigh (approximately 10 cm above the lateral epicondyle), on the sternum (approximately 5 cm under the clavicula), and on the dominant upper extremity (approximately 5 cm under the acromion) (Fig. 1). Following a standardized activity protocol, participants were asked to complete an obstacle course based on chosen activities. The activities were performed right after each other with no interval for rest (Table 1). The trial was completed in the gym hall of each school, and the participants came in pairs from the same class. Accelerometer data were collected and processed using a standard algorithm to generate variables on duration and distance. Activity performance was videotaped with two tablets. Video observation was chosen as gold standard [12, 13] for the comparison to SENS to assess the concurrent validity.

**Table 1:** Standardized activities

Activity	Description	Duration/distance
Sitting (chair)	Seated on a standard chair (height 45 cm) with backrest	1 min
Walking (normal)	Self-selected speed	30 m
Walking (fast)	Fast walking	30 m
Lying (supine)	Supine position, lying on a plinth with head on pillow	1 min
Lying (side)	Lying on plinth on either left or right side with head on a pillow	1 min
Arm movement (sitting)	Bounce a balloon with dominant arm while sitting on a chair	1 min
Arm movement (standing)	Bounce a balloon with dominant arm while standing	1 min
No. of steps	Measured during walking at normal speed	30 m

### SENS accelerometer

SENS consists of a small waterproof sensor (45 × 21 × 5, 6 g) with a triaxial accelerometer registering acceleration in three axes at a 12.5-Hz sampling frequency. The SENS battery lifetime is 20 weeks, and a 14-day data storage capacity allows the system to save recorded data continuously if the sensor is beyond smartphone range. When the sensor is within 15 m of a smartphone, it connects wirelessly, and data are transmitted to the application every 10 min. The raw accelerometer data are automatically transferred to a secured web server via the smartphone’s network connection. Raw acceleration is calculated as the average magnitude of movement based on high-pass filtered 3-axis accelerometer measurement at 12-Hz sampling frequency. Data are sampled in 5-s epoch sizes at a sampling frequency of 12.5 Hz and automatically categorized into time spent in the predefined categories, e.g., walking, sitting/lying, or number of steps [14].

### The protocol

The standardized activities were chosen to examine the sensor’s ability to classify sedentary behavior and physical activities, including arm movements (Table 1). The chosen activities presented in our study are relevant, basic, and important activities, and are to some extent similar to activities performed in comparable validity studies [15–17]. Furthermore, the activities were chosen based on the predefined categories in the SENS algorithm [14]. All participants performed the activities in the same order and were guided through the obstacle course by one of the authors (C. M.). The other author recorded the time-limited activities using a stopwatch and noted the exact starting and finishing time for the entire obstacle course on a sheet with a unique ID number for each participant. The video data were analyzed separately for each activity to calculate the time spent in the respective activities. The number of steps was compared with video-observed slow-motion steps during walking. Data from SENS were sampled at 5-s intervals, whereas the total time spent in the predefined categories and the number of steps were calculated based on the entire activity duration, including the initial and completed 5-s interval for the activity.

### Data analysis

All scalar descriptive data were tested for normal distribution and are presented as means with standard deviations (SD). Categorical variables are presented as frequencies with percentages. The walking and fast walking speed were calculated in meters per second from the observed data and are presented as mean (SD). The paired *t*-test was performed for all variables to determine if systematic differences existed between the two methods, and the agreement between SENS and video observation is illustrated in Bland–Altman plots and 95% limits of agreement (LoA) [18, 19]. Concurrent validity was further evaluated using the intraclass correlation coefficient (ICC_2.1_) as appropriate for evaluating rater-based clinical assessment methods [20], and was expressed as standard error of measurement (SEM and SEM%) and minimal detectable change (MDC and MDC%). SEM is the square root of within-subject variance (i.e., the square root of the total variance, excluding between-subject variance). The MDC indicates the error associated with multiple completions of the same scale (e.g., when the same patient completes an activity-specific scale at initial evaluation and at discharge) [21, 22]. SEM and MDC were calculated to reflect the measurement error and variation between SENS and video observation. Based on categories to interpret reliability, ICC values < 0.5 were considered of poor validity; values between 0.5 and 0.75, moderate validity; values between 0.75 and 0.9, good validity; and values > 0.9, excellent [20]. The following measurement error values were chosen: < 10% = low, between 10 and 15% = acceptable, and ≥ 15% = high [8].

## Results

A total of 69 students were invited to participate of whom 38 were eligible for inclusion. A total of 31 students either did not want to participate or did not have a parent-signed consent form. Two participants were sick on the test day; thus, the total number of participants included in the analysis was 36. Among the included participants, 61% were female, and the mean (SD) age was 10.2 (2.3) years (Table 2).

**Table 2:** Participant characteristics, *N* = 36

Variables	*N* (%)	Mean (SD)
Sex *Girls* *Boys*	22 (61.1%) 14 (38.9%)	
1st grade	13 (36%)	
3rd grade	9 (25%)	
6th grade	14 (39%)	
Age (years)		10.2 (3.2)
Weight (kg)		38.4 (11.9)
Height (cm)		146.9 (15.7)
Body mass index (kg/m^2^)		17.4 (2.7)

### Comparison of thigh sensor recordings

**Fig. 2:**
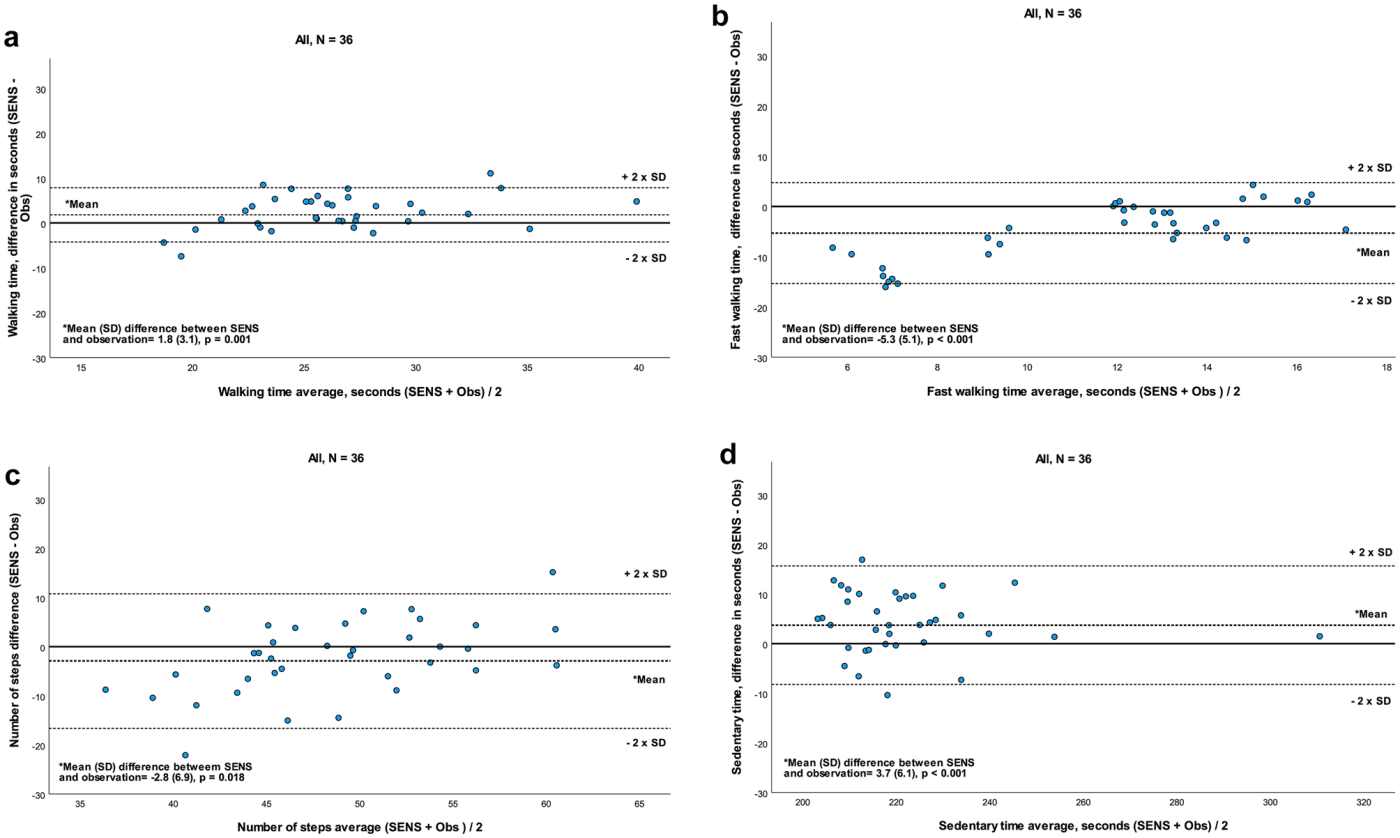
**a–d** Bland–Altman plot for agreement between SENS motion^®^ and video observation for walking time, fast walking time, sedentary time, and number of steps, recorded by a sensor placed on the thigh

The recordings from the thigh sensor provided data on walking time, fast walking time, sedentary time, and number of steps. For all variables, a systematic difference (*p* ≤ 0.018) for the comparison between SENS and observation was seen (Fig. 2a–d; Table 3). Strong agreement was observed for walking time and sedentary time with only a small LoA spread (Fig. 2a and d). The self-selected speed during walking time was 1.3 (0.2) m/s. One outlier was observed for sedentary time with a mean value significantly larger than the rest of the dataset. This participant was lying for a longer time than the others, while some technical issues were dealt with during data collection, which explained the outlier. Agreement for steps was moderate, and for fast walking time, greater variance in the differences was found with the LoA revealing a weak agreement for comparison between SENS and observation (Fig. 2b, c). Self-selected speed for fast walking time was 2.3 (0.3) m/s.

**Table 3:** Concurrent validity assessed by SENS motion^®^ and video observations, *N* = 36

Variables	Mean (SD) SENS	Mean (SD) Observation	Mean (SD) SENS and observation	Mean (SD) Difference (s)	*P* value for difference	ICC_2.1_ (95% CI)	SEM (SEM%)	MDC (MDC%)
Walking time (s)^a^	27.4 (5.5)	25.6 (4.1)	26.5 (4.9)	1.8 (3.1)	0.001	0.747 (0.463–0.877)	2.4 (9)	6.7 (25)
Fast walking time (s)^a^	9.1 (5.8)	14.4 (1.6)	11.7 (5.0)	− 5.3 (5.1)	< 0.001	0.152 (−0.089–0.412)	5.5 (47)	15.2 (130)
No. of steps^a^	47.2 (8.5)	50.0 (5.5)	48.6 (7.3)	− 2.8 (6.9)	0.018	0.500 (0.210–0.709)	5.2 (11)	14.4 (30)
Sedentary time (sitting/lying) (s)^a^	223.8 (19.8)	220.1 (19.8)	221.9 (19.7)	3.7 (6.1)	< 0.001	0.937 (0.828–0.973)	4.9 (2)	13.6 (6)
Sitting time (s)^b^	73.0 (15.0)	82.9 (10.6)	78.0 (13.9)	− 9.9 (10.6)	< 0.001	0.527 (0.027–0.779)	9.6 (12)	26.6 (34)
Lying time (s)^b^	136.1 (15.8)	137.4 (16.3)	136.7 (15.9)	− 1.3 (5.6)	0.169	0.938 (0.882–0.968)	4.0 (2)	11 (5)
Time with arm movement (s)^c^	128.6 (6.2)	123.3 (3.9)	125.9 (5.8)	5.3 (4.0)	< 0.001	0.469 (−0.092–0.771)	4.2 (3)	11.6 (9)
Time with no arm movement (s)^c^	68.4 (19.1)	80.5 (12.5)	74.4 (17.6)	− 12.1 (12.8)	< 0.001	0.559 (0.034–0.801)	11.7 (16)	32.4 (44)

SEM and MDC indicate measurement error (in s) at group and individual levels, respectively

*ICC* intraclass correlation coefficient, *SEM* standard error of measurement (SD of mean [both assessments] × √1 − ICC), *MDC* minimal detectable change (SEM × √2 × 1*.*96), *SEM%* 100 × SEM/mean, *MDC%* 100 × MDC/mean

^a^Assessed by a sensor on the thigh

^b^Assessed by a sensor on the sternum

^c^Assessed by a sensor on the upper extremity

A good to excellent ICC was seen for walking time (ICC = 0.75) and sedentary time (ICC = 0.94). For number of steps, ICC was moderate (ICC = 0.5), and for fast walking time, ICC was poor (ICC = 0.15). For walking time and sedentary time, group-level measurement error (SEM) was low (2–9%). SEM reached an acceptable level for number of steps (11%) and was high for fast walking time (47%). The corresponding individual-level measurement error (MDC) for all variables was high (25–130%) except for the comparison between SENS and observation for sedentary time, which was lower (6%) (Table 3).

### Comparison of sternum sensor recordings

**Fig. 3:**
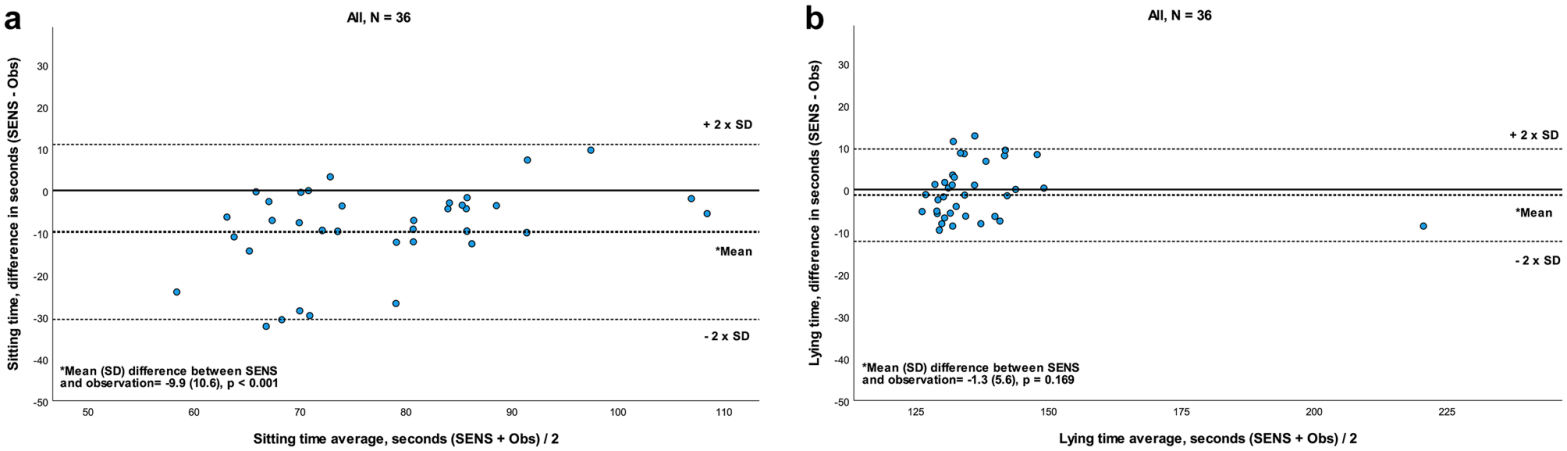
**a** and **b** Bland–Altman plot for agreement between SENS motion^®^ and video observation for sitting and lying time, recorded by a sensor placed on the sternum

The recordings from the sternum sensor provided data on sitting and lying time. A systematic difference (*p* < 0.001) for the comparison between SENS and observations was seen only for sitting time (Fig. 3a; Table 3) and not for lying time (*p* = 0.169) (Fig. 3b; Table 3). For sitting time, LoA range showed a greater variance and revealed a moderate agreement between SENS and observation (Fig. 3a). A strong agreement existed for lying time with a small LoA range. The outlier that appeared for lying time was the same participant as for sedentary time. The ICC for sitting time was moderate (ICC = 0.53) and the corresponding SEM only reached an acceptable level (12%). For lying time, an excellent ICC was seen (ICC = 0.94) with a low SEM (2%) and MDC (5%) (Table 3).

### Comparison of upper extremity sensor recordings

**Fig. 4:**
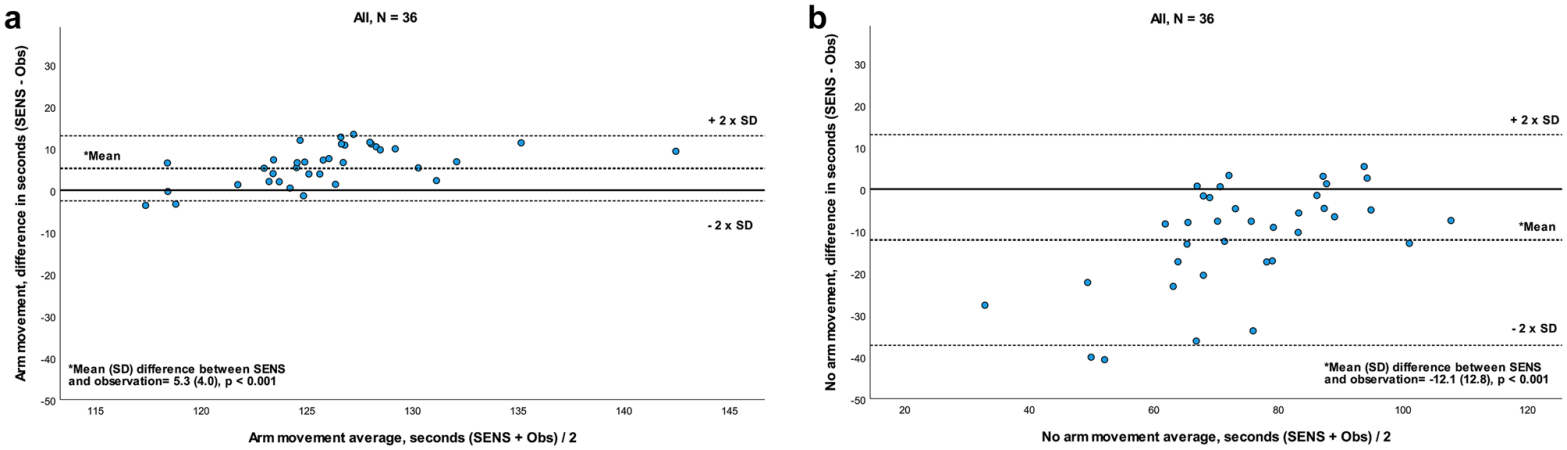
**a** and **b** Bland–Altman plot for agreement between SENS motion^®^ and video observation for time with arm movement and no arm movement, recorded by a sensor placed on the upper extremity

The recordings from the upper extremity sensor provided data on time with and without arm movement. For the comparison of SENS and observation, a systematic difference (*p* < 0.001) was seen for both time with arm movement and time with no arm movement (Fig. 4a and b; Table 3). For time with arm movement, the LoA showed a small spread in variance and that SENS overestimated in 89% of the cases, showing moderate agreement between SENS and observation (Fig. 4a). A moderate agreement also existed for time with no arm movement (Fig. 4b). For time with arm movement, the ICC value was poor (0.47), whereas the corresponding measurement error was low at both group level (SEM) and individual level (MDC) (from 3 to 9%). ICC for time with no arm movement was moderate (ICC = 0.56), and the level of SEM (16%) and MDC (44%) showed a high error rate for comparison between SENS and observation (Table 3).

## Discussion

This study aimed to evaluate the validity of SENS motion^®^ compared with video observations for monitoring physically active time and sedentary time in healthy children and adolescents. An overall moderate to strong agreement between SENS and observation in all variables except for fast walking time supported the concurrent validity of the SENS motion^®^ system. The ICC for comparison between SENS and observation was moderate to good for walking time, number of steps, sitting time, and time without arm movement, whereas it was excellent for sedentary and lying time. The corresponding evaluation of the group-level measurement error (SEM) was low for walking, sedentary, lying time, and time with arm movement. An acceptable level was reached for steps and sitting time.

### Walking time, fast walking time, and number of steps

Our result on walking time revealed a strong agreement between SENS and observation, which is consistent with the findings of several previous studies evaluating the validity of comparable thigh worn accelerometers in children and finding a correspondingly strong agreement in walking time [15, 16, 23]. A systematic review identified only a 68% accuracy in accelerometers placed on the hip in children during light intensity activities equivalent to walking at a regular pace [17]. Thus, accelerometers placed on the thigh rather than the hip may possibly provide greater accuracy in detection of walking in children and adolescents.

Our walking time results ran contrary to those of a recent study on elderly patients, where only a moderate agreement was seen between SENS and observation [8]. The divergent results may possibly be explained by walking pace. It was demonstrated that accelerometers may be challenged when measuring slow walking and fast [8, 24, 25]. Our study corroborated this finding regarding fast walking time. However, it seems that walking duration is to some extent also relevant.

In our study, the mean value for fast walking time was 14.4 s, which may be considered a short duration. Another study with a longer measurement time found higher accuracies during fast walking on a treadmill [15]. In our study, the participants walked at a self-selected fast walking pace, and a misclassification may possibly have occurred, since SENS possibly categorized fast walking as running.

In our study, the moderate agreement of steps was measured during an average time period of 25.6 s, whereas previous studies in children have reported a high accuracy for steps collected in > 2 min [23, 26]. Based on the results, it should be noted that measuring for short periods of time or over distances may, to some extent, reduce the validity of SENS.

### Sedentary time, lying time, and sitting time

According to the literature, accelerometry detects sedentary behavior in children with high accuracy [15–17, 27], which our results confirmed with a low measurement error at both group and individual level. However, the sensors placed on the thigh failed to differentiate between the sitting and the lying position. This differentiation may be elicited by placing the sensor on the sternum. Our findings on lying time were similar to those of previous studies, since the placement on the sternum or lower back is designed to detect this position and to differentiate it from sitting/standing [8, 15, 28].

A misclassification regarding sitting time might have occurred in the present study, where a moderate agreement was revealed, since the sensor placed on the sternum did not distinguish sitting from standing. The distinction between sitting and standing is relevant since standing potentially has a greater impact on physical activity level [29] as it requires greater muscle activity and a higher energy expenditure than sitting. Furthermore, this would provide the opportunity to quantify the time out of bed, which may be of high relevance in hospital settings. For this purpose, a dual monitor system with an additional sensor placed on the sternum or back is required. The SENS motion^®^ system has been found to be valid for distinguishing between time in and out of bed in elderly patients [8], but the system has not yet been tested in children. To the best of our knowledge, validity studies of dual systems in children are still limited [15], and may be a relevant area for future research.

### Time with and without arm movement

Besides hip- and thigh-worn devices, accelerometers on the wrist have been widely used for measuring and quantifying physical activity and sedentary behavior [30]. Wrist-worn devices have been demonstrated to be valid in assessing physical activity in children [31–33] and adolescents [34]. However, consensus seems to be lacking with respect to sensor placement on the upper extremity for assessing physical activity or movements. Previous studies placed accelerometers on the forearm [35], elbow [36], triceps [37], and upper arm [38]. In the present study, SENS devices were placed on the proximal part of the upper extremity. We chose this location to reduce noise from, e.g., hand movements. An underestimation of time without arm movements with a corresponding overestimation of time with arm movements was seen. This was presumably caused by the algorithm that was developed to detect lower extremity acceleration. Thus, less acceleration from the upper extremity is required for the accelerometer to register a movement, which has also been reported previously [31].

An algorithm developed and calibrated for upper extremity acceleration would, in future research, potentially improve the ability of SENS to detect such movements. This assessment is currently explorative, and as validity studies lack standardization, comparability remains a challenge [39, 40]. However, in the present study, the moderate agreement between SENS and observation and the low measurement error at both group and individual level for arm movement seems promising. Assessing physical activity is primarily done by measuring acceleration in lower extremities, but accelerometry of measurement of the upper extremities may potentially contribute to assessment methods for upper limb treatment and rehabilitation in addition to providing a further means of quantifying physical activity in children unable to use their lower extremities due to disabilities or diseases.

### Strengths and limitations

A major strength of this study was the method employed in which video observations were used as the reference criteria. This design reduced measurement errors for the registration of time and number of steps [13, 40]. This study also had some limitations. Due to the relatively small sample size, our findings should be interpreted with caution [41]. The information detailing how the accelerometer categories were established is not published in a medical publication, but the raw accelerometer data and technical details are available from SENS [14]. Furthermore, the limitations of this study included a relatively short time span during which the activities were assessed. However, this may possibly have allowed us to reflect in a more natural manner the variation in a child’s activity behavior. Additionally, it would be appropriate to examine SENS motion^®^ in a free-living condition, which would have encompassed a wider variation in children’s natural movement pattern, and to validate the system in relation to activities of daily living. However, we adopted a pragmatic approach for this study as observation provided our reference criteria.

## Conclusion

Compared with video observations, SENS motion^®^ seems to be valid for detecting sedentary behavior and specific standardized types of physical activity in a small group of healthy children and adolescents. A good to strong agreement was seen for the comparison of SENS and video observation for walking, sedentary, and lying time, and revealed by a good to excellent ICC (0.75–0.94). The corresponding group level measurement error was low, including time with arm movement (2–9%). The agreement in number of steps and time with and without arm movements was moderate, and ICC was poor to moderate (0.47–0.56). The results on arm movements seem promising; however, further studies are warranted to examine this explorative area.

## Abbreviations

MVPA: Moderate-to-vigorous physical activity
ICC: Intraclass correlation coefficient
SEM: Standard error of measurement
MDC: Minimal detectable change
LoA: Limits of agreement
SD: Standard deviation
